# Effects of γ-Ray Irradiation on the Radial Structure Heterogeneity in Polyacrylonitrile Fibers during Thermal Stabilization

**DOI:** 10.3390/polym10090943

**Published:** 2018-08-24

**Authors:** Wei Dang, Jie Liu, Xiangyu Huang, Jieying Liang, Chunhua Wang, Peng Miao, Yongzhen An, Xiaoxu Wang

**Affiliations:** 1 Key Laboratory of Carbon Fiber and Functional Polymers, Ministry of Education, Beijing University of Chemical Technology, Chao-Yang District, Beijing 100029, China; buct_dangwei@163.com (W.D.); liangjy@mail.buct.edu.cn (J.L.); 2 Changzhou Institute of Advanced Materials, Beijing University of Chemical Technology, Changzhou 213164, China; wangchunhua2012@gmail.com (C.W.); pengmiao139@139.com (P.M.); anyongzhen1991@163.com (Y.A.); 3 SINOPEC Shanghai Petrochemical Company Limited, 48 Jinyi Road, Jinshan District, Shanghai 200540, China; huangxy.shsh@sinopec.com

**Keywords:** polyacrylonitrile fibers, radial structure heterogeneity, γ-ray irradiation, thermal stabilization

## Abstract

The radial structural heterogeneity of thermally-stabilized polyacrylonitrile (PAN) fiber is considered to be a limiting factor affecting the mechanical properties of the resulting carbon fibers. In this study, we demonstrate that a low-dose (60 kGy) γ-ray irradiation pretreatment can effectively mitigate the radial structural heterogeneity of PAN fibers after thermal stabilization. The characterization results indicate that low-dose γ-ray irradiation only affects the physical structure of PAN through decreasing its crystalline size and crystallinity, rather than inducing chemical cross-linking and/or intramolecular cyclization. It is proposed that an increased amorphous region in PAN fibers prompted by low-dose γ-ray irradiation can facilitate oxygen diffusion from skin to core during stabilization, which results in the increased structural homogeneity of stabilized PAN fibers.

## 1. Introduction

Carbon fibers made from polyacrylonitrile (PAN) precursor fibers have attracted much attention due to their superior mechanical properties and outstanding chemical stability [1,2]. The commercial carbon fibers possess a tensile strength of 2–7 GPa and a tensile modulus of 200–900 GPa [3]. However, based on the calculation of C–C bonding strength, the theoretical tensile strength and modulus of carbon fibers are 180 GPa and 1000 GPa, respectively [4,5]. The significant difference between theoretical and practical mechanical properties is due to the presence of structural imperfections in carbon fibers, such as defects and structural heterogeneities [5,6].

The manufacture of carbon fibers typically involves three steps: Spinning of PAN precursor fibers, thermal stabilization, and carbonization. The thermal stabilization process is the most critical step during which the linear PAN chains convert to a thermally stable ladder structure; this step ensures efficient conversation of polymer to carbon with a high structural integrity and carbon yield [7]. Stabilization occurs in an oxidative environment (such as in air) and is a diffusion-controlled process [5,8]. Oxygen participates in the thermal stabilization through dehydrogenation and oxidation reactions and can facilitate intermolecular crosslinking [9]. However, the oxidation on the outer region of the fiber would generate a dense layer and hinder further diffusion of oxygen into the inner region [10,11]. The consequence is that the outer region of PAN fiber has been well-stabilized while the inner region has not, which corresponds to the formation of the skin-core structure and the stabilized fibers are therefore structurally inhomogeneous. This radial structural heterogeneity will further transfer to the final carbon fibers as structural defects and deteriorate their tensile properties [12,13].

γ-ray irradiation has been successfully applied for the pretreatment of PAN fibers due to its powerful penetrability [14,15,16,17,18,19]. It is shown that γ-rays can initiate radical induced intermolecular cross-linking and intramolecular cyclization of PAN polymer chains to form a ladder structure [14,15]. As thermal stabilization is an exothermic process, pretreating PAN fibers using γ-ray irradiation can lower the reaction onset temperature and mitigate the evolution of heat [15]. Based on these effects, some efforts have been devoted to utilizing γ-ray irradiation to decrease thermal stabilization time and improve stabilization efficiency [17]. Among these studies, a high irradiation dose (200–2000 kGy) is typically applied to PAN fibers in order to achieve enough conversion of the PAN molecules. 

It is noteworthy that γ-rays can penetrate through the PAN fiber without significant energy loss. Thus, it can be assumed that the cross-linking and cyclization reactions induced by γ-ray irradiation take place homogeneously throughout the whole PAN fiber, and the radial structure heterogeneity of PAN fibers after stabilization can therefore be improved. However, research on the improvement of the radial structure heterogeneity of PAN fibers using γ-ray irradiation is quite limited.

In this study, PAN fibers are pretreated with low-dose γ-ray irradiation and then processed through a traditional oxidative stabilization treatment. The effect of irradiation on the radial heterogeneity of stabilized PAN fibers is qualitatively and quantitatively analyzed using optical microscopy and energy-dispersive X-ray spectroscopy, respectively. The effect of γ-ray irradiation on the chemical and physical structures of PAN fibers is studied using DSC, FT-IR, ^13^C-NMR, and XRD. The mechanism of the irradiation effect was also discussed based on a systematic investigation of the structural evolution of PAN fibers.

## 2. Materials and Methods

### 2.1. γ-Ray Irradiation and Stabilization of PAN Fibers

The PAN precursor fibers (wet-spun, 12,000 filaments/tow) were supplied by SINOPEC Shang Hai Petrochemical Company Limited (Shanghai, China). The PAN fibers were irradiated by ^60^Co γ-rays in air at room temperature, and a low dose rate of 4.3 kGy/h was applied in order to avoid local temperature rises due to the thermally insulating nature of the polymers [20,21,22,23]. The total dosage was 60 kGy and was achieved by controlling the irradiation time. The irradiated PAN fiber was denoted as i-PAN. Both untreated PAN fibers (PAN) and irradiated PAN fibers (i-PAN) were stabilized continuously through four oxidizing furnaces at temperatures from 220 to 265 °C for 36 min. Detailed stabilization profiles can be found in the Supporting Information, Table S1. The stabilized PAN and i-PAN fibers were denoted as SFs and i-SFs, respectively. Carbonization was conducted from 350 to 1300 °C in a nitrogen atmosphere for 3 min.

### 2.2. Characterizations on the Fiber Samples

The density of fiber was measured at 23 ± 0.1 °C by using a density gradient tube filled with carbon tetrachloride and *n*-heptane.

Differential Scanning Calorimetry (DSC, METTLER Toledo DSC-822, Mettler-Toledo, Schwerzenbach, Switzerland) was used to investigate the exothermic properties of PAN and i-PAN fibers. A DSC test was performed at a heating rate of 5 °C/min under an air atmosphere, and the range of temperatures was 40–400 °C.

Fourier Transform Infrared Spectrometer (FT-IR, Nicolet 8700, Thermo Fisher Scientific, Waltham, MA, USA) and ^13^C Nuclear Magnetic Resonance (^13^C-NMR, AV300, Bruker, Zurich, Switzerland) were used to characterize the chemical bonds of PAN and stabilized PAN fibers.

X-ray diffraction (XRD, D/max-2550 PC, Rigaku Corporation, Tokyo, Japan) was used to characterize the microstructure of PAN fibers, including crystallinity and orientation characterized by 2-theta scanning and azimuth scanning respectively.

The skin-core structure of oxidized PAN fibers was characterized by the Optical Microscope (OM, Shanghai Optical Instrument Factory, Shanghai, China). The fiber samples were embedded in resin, and then cut into slices with a 500 nm thickness using a Leica EM UC7 microtome (Leica Mikrosysteme GmbH, Wien, Austria).

The relative oxygen content of fibers in the cross-section was characterized by Energy Dispersive Spectrometer (EDS, Genesis 60, EADX Inc., Mahwah, NJ, USA) with Scanning Electron Microscope (SEM, S-4700, Hitachi Limited, Tokyo, Japan). The element content within the fiber was characterized using an Elemental Analyzer (Thermo Fisher Scientific, Waltham, MA, USA).

## 3. Results

The thermal stabilization is conducted on a continuous pilot production line with four temperature zones, and the degree of stabilization was controlled by adjusting the temperature of each furnace. The density change of PAN fiber during stabilization was typically used to find the optimal stabilization conditions, and the typical density of the stabilized PAN fiber is 1.36–1.38 g/cm^3^ [1]. The densities of stabilized PAN fibers (SF) and stabilized i-PAN fibers (i-SF) are 1.3639 and 1.3728 g/cm^3^, respectively, which confirms that the stabilization conditions in this study were adequate.

During the thermal stabilization process, the color of the PAN fibers progressively changes to yellow, brown, and black as heating is continued; this discoloration is accelerated by the presence of oxygen from the air [24]. The color formation at elevated temperatures is characteristic of acrylic fibers and is attributed to the conjugation bonds and aromatic structures generated by the oxygen-induced reaction of dehydrogenation [24]. In view of this effect, the degree of stabilization reaction can be traced by the color transformation of the PAN fibers, and the optical microscopy of a thin-sliced PAN fiber is one typical method to characterize the radial structure heterogeneity [11,13].

**Figure 1:**
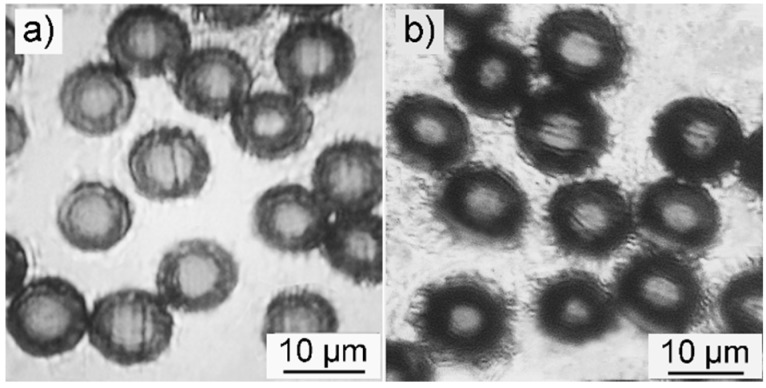
Optical microscopy images showing the cross-section of sliced (**a**) stabilized PAN fibers (SFs) and (**b**) stabilized i-PAN fibers (i-SFs). Note: Polyacrylonitrile (PAN) fibers are untreated and i-PAN fibers are irridated polyacrylonitrile fibers.

Figure 1a,b shows the cross-section views of SFs and i-SFs, respectively. Both pristine and irradiated PAN fibers demonstrate a core-shell structure after stabilization in air. The i-SFs have thicker and darker shells compared with SFs. The darker color indicates that i-SFs have a higher degree of stabilization [13]. The thicker shell of i-SFs can presumably be explained by the deeper penetration of oxygen into the core of the fiber during thermal stabilization. While the optical microscopy can provide intuitive information about the radial structure of stabilized PAN fibers, more evidence comes from the quantitative analysis of oxygen content distribution on the cross-section of the fiber.

**Figure 2:**
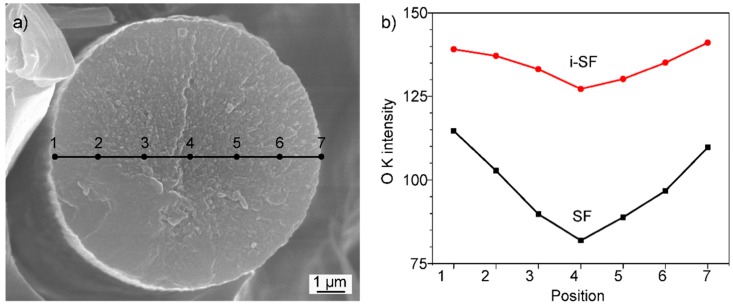
(**a**) SEM image of the cross-section of a stabilized PAN fiber illustrating the mapping lines and positions for oxygen content distribution using EDS analysis; (**b**) oxygen content of SFs and i-SFs at specific line scan position.

The oxygen distribution across the cross-section of stabilized fibers is compared for SFs and i-SFs based on the scanning of oxygen using a focused ion beam (FIB) of energy-dispersive X-ray spectroscopy (EDS). As shown in Figure 2, i-SFs show a more uniform distribution of oxygen than SFs. These results are in agreement with the optical microscopy that pretreatment with low-dose γ-ray irradiation can successfully improve the structural uniformity of the stabilized PAN fibers. Meanwhile, the overall oxygen content of i-SFs is higher than SFs, as shown in Figure 2b. Elemental analysis results, see the Supporting Information, Table S2, show that the oxygen content in PAN fibers is similar before and after irradiation. Thus, the increase in the oxygen content in i-SF can be attributed to the facilitation of the stabilization reaction in the irradiated PAN fibers. In order to elucidate this effect, a systematic investigation of the structural evolution of PAN fibers after irradiation and thermal stabilization is performed.

**Figure 3:**
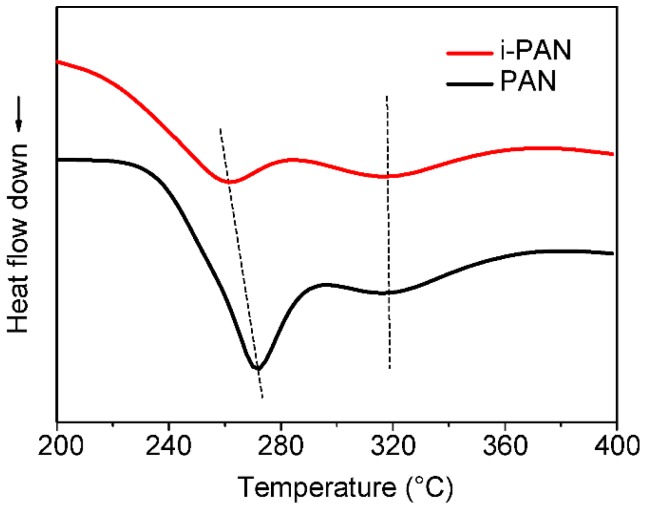
DSC curves of PAN and i-PAN fibers measured in an air atmosphere.

Differential scanning calorimetry (DSC) is first employed to study the thermal behavior of both PAN and i-PAN fibers, and the results are shown in Figure 3. The second exothermic peak at ~319 °C is known as the oxidation reaction when PAN fibers are stabilized in air [25]. There is no apparent difference of peak position and peak pattern between PAN and i-PAN fibers, which indicates that low-dose irradiation does not facilitate the oxidation reaction of PAN fibers in the subsequent thermal stabilization process. Based on the elemental analysis, the oxygen content of i-SF and SF is 11.3% and 9.2%, respectively. Since the oxidation rate was suggested to be unaffected by irradiation, the higher oxygen content in i-SF may be due to the higher diffusion rate of oxygen into the irradiated PAN fibers.

The onset temperature of the exothermic reaction noticeably decreased for irradiated PAN fibers. Meanwhile, the exothermic peak at 272.6 °C for untreated PAN fibers shifted to a lower temperature as the fibers were irradiated with γ-rays. Accompanied by the temperature shifts, the peaks also broadened after irradiation treatment. It is well known that the first exothermic peak centered at 272.6 °C is assigned as the cyclization reaction of PAN molecules [25]. The shifted and broadened peak pattern indicate that the exothermic cyclization reaction of PAN was facilitated and mitigated when the fibers were treated with γ-ray irradiation. Similar observations have been reported by several research groups and they attribute this to irradiation-induced cyclization of the PAN through a radical mechanism, which means that the PAN polymer chains were partially reacted and cyclized after irradiation, as verified by the FT-IR analysis [14,15,16,17,18,19,20].

**Figure 4:**
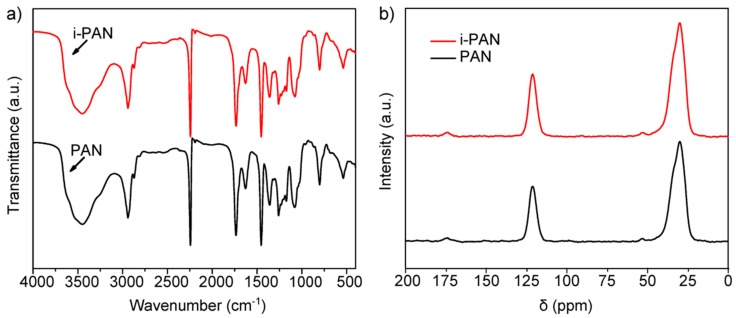
(**a**) FT-IR spectra; and (**b**) ^13^C-NMR spectra of PAN and i-PAN.

To verify if the observed dissimilar exothermic behavior of PAN and i-PAN is the result of a chemical reaction induced by irradiation, FT-IR is conducted to trace the changes of the chemical structures on both PAN and i-PAN samples and is shown in Figure 4a. In general, as the cyclization reaction proceeds, more nitrile groups (C≡N) would convert to a cyclized structure (C=N), which corresponds to the decreased peak intensity at 2243 cm^−1^ and increased peak intensity at 1580–1620 cm^−1^ [26]. PAN fibers irradiated with high-dose gamma-rays would exhibit a reduced peak intensity of C≡N groups and increased peak intensities of C=O, C=N, and C=C groups. Interestingly, there is no observable difference between the FT-IR spectra of non-irradiated and irradiated PAN fibers. The results indicate that the intermolecular cross-linking and/or intramolecular cyclization reaction are not initiated by low-dose γ-ray irradiation, which is in contrast with previous FT-IR analyses based on high-dose irradiated PAN fibers [27]. Such low-dose irradiation with an ultra-slow irradiation rate may not activate the chemical reaction of PAN.

In order to justify the FT-IR results and further track the chemical reaction, ^13^C-NMR analysis is applied to both PAN and i-PAN fibers, as shown in Figure 4b. Both samples show aliphatic carbon signals at 33 ppm and a C≡N signal at 121 ppm, which are characteristic of PAN [28]. Meanwhile, a weak C=O signal at 175 ppm is present in both samples, indicating the PAN fiber used in this study is a copolymer of acrylonitrile (AN) and other comonomers, such as carboxylic acid and itaconic acid. Previous studies on the stabilization of PAN reveal that new peaks would appear in the 110 and 155 ppm region on the ^13^C-NMR spectra due to the formation of C=C and C=N after cyclization and dehydrogenation reactions [29]. However, in this study, both the peak position and peak area are identical between PAN and i-PAN, and there is no new peak detected. This confirms that no chemical reaction is induced by low-dose irradiation.

**Figure 5:**
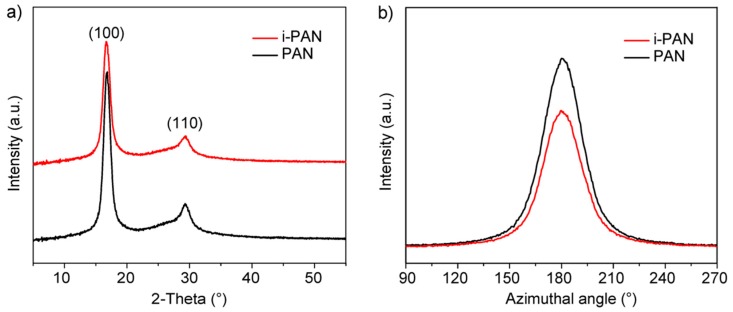
XRD patterns of PAN and i-PAN fibers with (**a**) 2-theta scanning; and (**b**) azimuth scanning at 16.7° for the (100) lattice plane.

There is no observable difference of the surface morphology between PAN and i-PAN, as revealed by the SEM images in Figure S2 (Supporting Information). Since the chemical structure and surface structure of PAN fibers are not affected by irradiation, it is natural to assume that the exothermic behavior of irradiated PAN fibers is due to the transformation of the physical stacking structure of the PAN chains. XRD analysis is employed to characterize the crystalline structures of PAN and i-PAN fibers and is shown in Figure 5.

In Figure 5a, the diffraction peaks centered at 2θ = 16.8° and 29.2° correspond to the (100) and (110) crystallographic planes of the PAN hexagonal lattice, respectively. The crystallinity (*X*_c_), and orientation index (*φ*) are calculated based on the XRD patterns and the results are listed in Table 1. The fitting (shown in Figure S1) and calculation method can be found in The Supporting Information. According to the morphological model of PAN fiber established by Warner, there are two aggregated structures of PAN chains in fibers in the form of quasicrystals and amorphous regions (i.e., ordered and disordered regions) [30]. As shown in Table 1, the orientation of PAN quasicrystals was retained after irradiation, which indicates that the stacking structure of the PAN crystallites was unchanged. On the other hand, the crystallinity of PAN fibers is noticeably decreased after irradiation, which indicates that the outer layer of PAN quasicrystals becomes amorphous as the result of low-dose irradiation.

The change of the PAN stacking structure also affects the mechanical properties of the PAN fibers. The mechanical properties of PAN and i-PAN fibers are measured based on single filament analysis. A total of 50 fibers were tested for each sample, and the tensile properties are listed in the Supporting Information, Table S3. The tensile strength of PAN fiber decreased after irradiation, and the rupture elongation increased. This can be explained by the increased amorphous region in irradiated PAN fiber, since the crystalline region of PAN is much more rigid than the amorphous region.

## 4. Discussion

**Figure 6:**
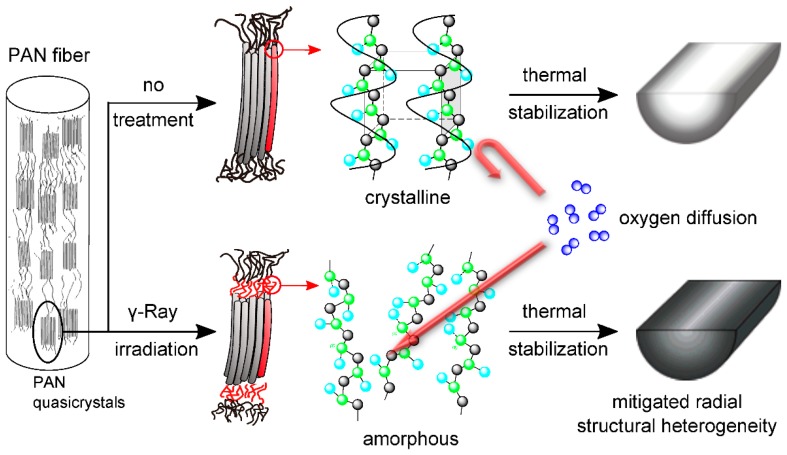
Schematic representation of the transition of PAN stacking structure from quasicrystal to amorphous after γ-ray irradiation and its effect on oxygen diffusion.

It is established that the cyclization reaction takes place preferentially in the amorphous regions and spreads to the quasicrystals [27,31]. Thus, the previous results on the facilitated exothermic cyclization reaction of i-PAN revealed by the DSC curves can be explained by the increased amount of amorphous region within the PAN fiber. The oxidation reaction of PAN during stabilization is a diffusion-controlled process [8,32]. As PAN polymer chains in the quasicrystals are more closely packed, the oxygen will diffuse easier within the amorphous region than in the crystalline region. As illustrated in Figure 6, for irradiated PAN fibers with higher amorphous contents, the oxygen can diffuse further into core of the fiber during the stabilization process, therefore enhancing the uniformity of the oxygen distribution, as well as the oxygen content, in stabilized PAN fibers.

Carbon fibers based on PAN and i-PAN fibers are prepared after stabilization and carbonization, and they are denoted as CFs and i-CFs, respectively. The tensile properties of both CF and i-CFs are listed in the Supporting Information Table S4. The i-CFs have a tensile strength of 3.03 GPa, which is slightly higher than that of 2.85 GPa for CFs. Meanwhile, the tensile modulus of both CF and i-CF are around 240 GPa. The slightly higher tensile strength of i-CFs can be ascribed to the increased degree of graphitization, as suggested by the ratio of D-band and G-band (denoted as *R*-value) in the Raman spectra of CFs and i-CFs. The Raman spectra and the *R*-value of CFs and i-CFs are shown in the Supporting Information Figure S3 and Table S4, respectively. The increased radial structural homogeneity of i-SF may account for the increased degree of graphitization after the carbonization process. There is no obvious difference in the surface morphology between CFs and i-CFs, as shown in the SEM images provided in the Supporting Information, Figure S4.

Although the tensile properties of i-CF are lower than the commercial T300 grade carbon fibers, they have met the requirement of automotive-grade carbon fibers as suggested by the U.S. Department of Energy’s (DOE). The DOE’s targets for the mechanical properties of automobile-grade carbon fibers are a 1.72 GPa tensile strength and 172 GPa modulus [33]. It is worth noting that the stabilization time of 36 min in this study is noticeably shorter than the typical stabilization time of 60–90 min for producing commercial carbon fibers [1]. Taking into account the short stabilization time and the satisfactory tensile properties, the i-CFs are promising low-cost candidates for use in the automobile industry.

## 5. Conclusions

In conclusion, low-dose γ-ray irradiation on PAN fibers can successfully reduce their radial structure heterogeneity during thermal stabilization. Thermal and structural analysis of both pristine and irradiated PAN fibers revealed that, rather than initiating chemical reactions, low-dose γ-ray irradiation can partially transform PAN quasicrystals to amorphous structure. The increased amorphous content in irradiated PAN fibers can facilitate the diffusion of oxygen into the core, which accounts for the enhanced radial structure uniformity in γ-ray irradiated PAN fibers after thermal stabilization. Carbon fibers based on irradiated PAN fibers (i-CFs) have a tensile strength of 3.03 GPa and a tensile modulus of 242 GPa. Taking into account the short stabilization time and satisfactory tensile properties, the i-CFs are promising low-cost candidates for the automobile industry.

## Figures and Tables

**Table 1:** Structural parameters of PAN quasicrystals derived from XRD analysis ^a^.

Sample	*X*_c_ (%)	*φ* (%)
PAN	63.8	85.4
i-PAN	56.9	85.2

^a^ *X*_c_: crystallinity; *φ*: orientation index.

## Supplementary Materials

The following are available online at http://www.mdpi.com/2073-4360/10/9/943/s1, Figure S1: Multi-peaks fitting of XRD of both (a) PAN and (b) i-PAN fibers, Figure S2: SEM images of (a) PAN and (b) i-PAN fibers, Figure S3: Raman spectra of carbon fibers based on (a) i-PAN; and (b) PAN fibers, Figure S4: SEM images of CF and iCF, Table S1: Experimental parameters of thermal stabilization and carbonization, Table S2: Element contents of PAN and i-PAN fibers, Table S3: Tensile properties of PAN and i-PAN fibers, Table S4: Tensile properties and *R*-value of CFs and i-CFs with the corresponding coefficient of variance (CV).
