# Supplementary material for: Effects of γ-Ray Irradiation on the Radial Structure Heterogeneity in Polyacrylonitrile Fibers during Thermal Stabilization
Source: Polymers (Basel). 2018 Aug 24;10(9):943. doi: 10.3390/polym10090943 (PMC6403893; doi:10.3390/polym10090943)
Supplement: Supplementary file 1 [file polymers-10-00943-s001.pdf]

Supplementary Materials: Effects of γ-Ray Irradiation on the Radial Structure Heterogeneity in Polyacrylonitrile Fibers during Thermal Stabilization

Wei Dang, Jie Liu, Xiangyu Huang, Jieying Liang, Chunhua Wang, Peng Miao, Yongzhen An and Xiaoxu Wang

Characterizations on the fiber samples

X-ray diffraction. The 2-theta scanning ranged from 5–60° under a scanning rate of 10°/min and a scanning step of 0.02°. The diffraction curves were analyzed using MDI Jade 5.0, the interlayer spacing (d_{100}) and the crystallite sizes (L_a and L_c) were calculated by the Bragg equation and the Scherrer formula, respectively:

$$d_{100} = \frac{\lambda}{2\sin\theta} \quad (1)$$

$$L = \frac{K\lambda}{\beta\cos\theta} \quad (2)$$

where λ is the wavelength of X-ray from Cu (0.15418 nm), θ is the diffracted angle, β is the full width at half maximum (FWHM), and K is shape factor (normally 0.9 for L_c, and 1.84 for L_a).

The crystallinity (X_c) of PAN fibers was calculated using the Bell and Dumbleton method shown in the literature[18]:

$$X_c = \frac{A_c}{A_c + A_a} \quad (3)$$

where A_c and A_a are the area of peaks corresponding to crystalline and amorphous in the XRD fitted through MDI Jade.

The degree of orientation was determined by the azimuthal-scans at 2θ of 17° and calculated from the following equation:

$$\varphi = \frac{180\text{-}H}{180} \times 100\% \quad (4)$$

where ϕ is the orientation index and H is the full width at half maximum (FWHM) of the diffraction peak.

The fiber samples for FTIR, ^{13}C-NMR, and element content were prepared as powder and dried at 60 °C for 12 h to get rid of the interference of water in air.

A Raman spectrometer (RM200, English) was used to analyze the degree of graphitization. In the Raman spectra, the "D-band" centered at ~1340 cm^{-1} is related to the disordered turbostratic structures, and the "G-band" centered at ~1580 cm^{-1} is related to the ordered graphitic structures. The *R*-value, which is the intensity ratio of D-band and G-band, indicates the amount of structurally ordered graphite crystallites within the fiber; and the small *R*-value means there is a high degree of structurally ordered graphite crystallites.

A Lloyd DC-2 density gradient tube was adopted to measure the bulk density of the stabilized fibers and the carbon fibers.

The mechanical properties of carbon fibers were tested on the basis of an ASTM D4018–99 standard using INSTRON-5567 universal testing machine at a crosshead speed of 10.0 mm/min with a gage length of 150 mm. Eight specimens were tested for each type of carbon fiber. During the specimen preparation, each type of carbon fiber was wound onto a rectangular framework in parallel and the aligned fibers were impregnated into a mixed solution (the mass ratio of acetone, epoxy 618

resin and triethylenetetramine was set at 20:10:1). Then, samples were cured in an oven at 120 °C for 2 h.

Multi-peaks fitting of XRD

The crystalline structures of PAN and i-PAN were characterized through XRD. The calculation of crystallinity (X_c), crystallite width (L_a), crystallite stack height (L_c), and interlayer spacing ($d_{(100)}$) was made through the use of the Equations 1–3, and the parameters in the equations were obtained by the multi-peak fitting of XRD. Both multi-peak fittings of XRD of PAN and i-PAN were shown in Figure S1, in which the r^2 corresponding to fitting errors.

Table S1. Experimental parameters of thermal stabilization and carbonization.

Furnace Number	1	2	3	4	Low-Temperature Carbonization	High-Temperature Carbonization
Temperature/°C	220	237	253	265	400–700	1200–1350
Duration time/min	10	10	10	6	2	2
Draw ratio/%	0	0	−1	−1	10	−5
Tension/cN	1300	1900	2300	3000	1000	2100

Table S2. Element contents of PAN and i-PAN fibers.

Sample	N/%	C/%	H/%	O/%
PAN	23.9	64.7	5.7	5.7
i-PAN	23.9	65.0	5.8	5.3

Table S3. Tensile properties of PAN and i-PAN fibers.

Sample	Fiber Diameter/μm	Fibre Number/dtex	Tensile Strength/cN/dtex	Rupture Elongation/%
PAN	12.52	1.32	5.4	16.86
i-PAN	11.96	1.29	5.1	17.38

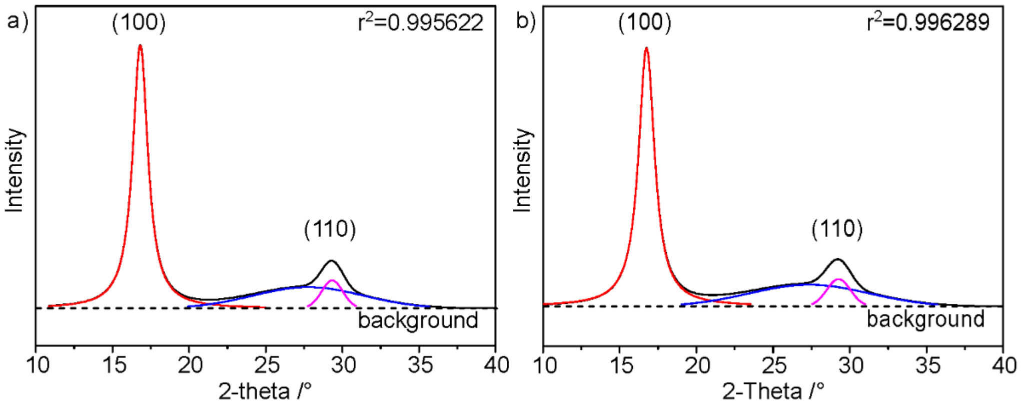

Figure S1. Multi-peaks fitting of XRD of both (**a**) PAN and (**b**) i-PAN fibers.

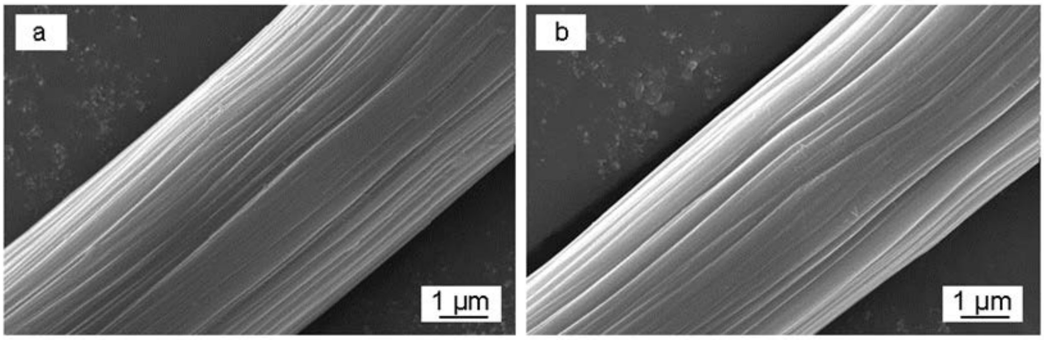

Figure S2. SEM images of (**a**) PAN and (**b**) i-PAN fibers.

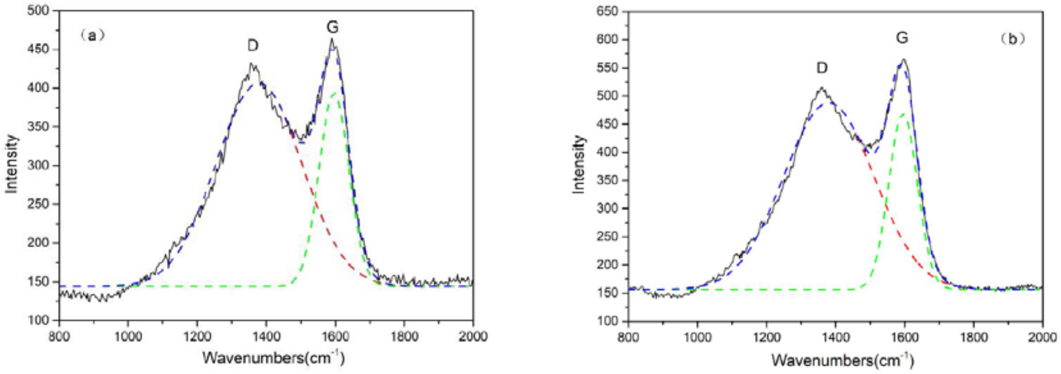

Figure S3. Raman spectra of carbon fibers based on (**a**) i-PAN; and (**b**) PAN fibers.

Table S4. Tensile properties and *R*-value of CFs and i-CFs with the corresponding coefficient of variance (CV).

Sample	Density (g/cm³)	Tensile Strength (GPa)	CV (%)	Young's Modulus (GPa)	CV (%)	Elongation (%)	CV (%)	*R*-value ($I_{(D)}/I_{(G)}$)
CFs	1.8017	2.85	0.93	237	2.01	1.60	1.59	1.35
i-CFs	1.7788	3.03	0.96	242	1.56	1.65	1.61	1.27

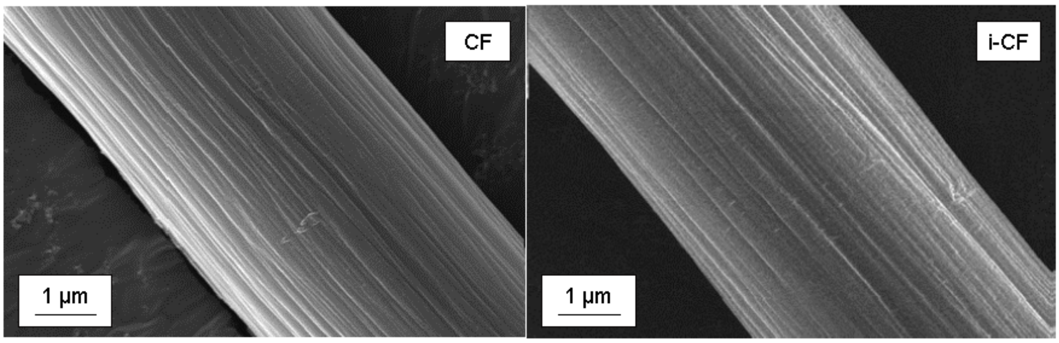

Figure S4. SEM images of CF and iCF.
